# OMN6 a novel bioengineered peptide for the treatment of multidrug resistant Gram negative bacteria

**DOI:** 10.1038/s41598-021-86155-9

**Published:** 2021-03-23

**Authors:** Shira Mandel, Janna Michaeli, Noa Nur, Isabelle Erbetti, Jonathan Zazoun, Livia Ferrari, Antonio Felici, Moshe Cohen-Kutner, Niv Bachnoff

**Affiliations:** 1 Omnix Medical Ltd., High-Tech Village, Givat-Ram Campus, 9270401 Jerusalem, Israel; 2 Evotec Anti-Infective, Department of Microbiology Discovery, Aptuit (Verona) S.R.L., an Evotec Company, via A. Fleming 4, 37135 Verona, Italy

**Keywords:** Antimicrobials, Peptides

## Abstract

New antimicrobial agents are urgently needed, especially to eliminate multidrug resistant Gram-negative bacteria that stand for most antibiotic-resistant threats. In the following study, we present superior properties of an engineered antimicrobial peptide, OMN6, a 40-amino acid cyclic peptide based on Cecropin A, that presents high efficacy against Gram-negative bacteria with a bactericidal mechanism of action. The target of OMN6 is assumed to be the bacterial membrane in contrast to small molecule-based agents which bind to a specific enzyme or bacterial site. Moreover, OMN6 mechanism of action is effective on *Acinetobacter baumannii* laboratory strains and clinical isolates, regardless of the bacteria genotype or resistance-phenotype, thus, is by orders-of-magnitude, less likely for mutation-driven development of resistance, recrudescence, or tolerance. OMN6 displays an increase in stability and a significant decrease in proteolytic degradation with full safety margin on erythrocytes and HEK293T cells. Taken together, these results strongly suggest that OMN6 is an efficient, stable, and non-toxic novel antimicrobial agent with the potential to become a therapy for humans.

## Introduction

Antimicrobial drugs are chemical substances having the capacity to kill or inhibit growth of microorganisms^1^. Antimicrobial drugs that are sufficiently nontoxic to the host are used to treat infectious diseases of humans, animals, and plants^2^. In order to lower or prevent lethal infectious diseases and maintain public health, new antimicrobial agents are required, especially against Gram-negative bacteria that stand for the majority of upcoming years' antibiotic-resistant threats^3^.

Antimicrobial peptides (AMPs) are part of the armament that organisms have developed to fight off pathogens, with many of them being produced by insects^4^. Although usually cationic, the primary structures of insect AMPs vary markedly^5^. Insects secrete antibacterial peptides to their hemolymph, as an innate defense against pathogenic infections^6^. Some insect species are capable of producing 10–15 antibiotic peptides^7^, each one being endowed with a completely different antibacterial range^4^.

Cecropins are small cationic peptides of 29–42 amino acids that are found in the Diptera and Lepidoptera orders^8^. They present strongly basic N-terminal regions, while their C-terminal parts contain long hydrophobic stretches and are with a neutral charge^9^. The spatial arrangement of Cecropins is characterized by two amphipathic α-helices that have the ability to penetrate the bacterial membrane^10^. This penetration is then followed by a loss of the membrane ionic gradient balance and finally to bacterial death^11^. The secondary structure of Cecropins is most likely linked to their membrane-disrupting activity, as proteins with amphipathic helices are often associated with membranes^6^.

Membrane-active peptides show channel-like conductivities across planar lipid bilayer systems as well as bilayer disruption^12^. These bilayer breaches deplete the affected organisms of their electrochemical gradients, leading to cell swelling due to a large water flow entrance, followed by a rapid increase in intracellular osmotic pressure and finally cell lysis and death^13^. This physicochemical mechanism of action has lower propensity to cause antimicrobial resistance^14,15^.

The antimicrobial peptides that present a certain interest for therapeutic use are those which display antibacterial activity, without showing any hemolytic or cytotoxic effect against healthy vertebrate cells^16^. In general, antibacterial peptides have a positive charge in order to bind to bacterial membranes, that normally present a negative charge^17^.

Cecropin A, a 37-residue peptide comprising only natural l-amino acids, is a strong example for Cecropin superiority as antimicrobial agents^18^. The major target site of Cecropin A is the bacterial membrane, with an antimicrobial effect likely based on its ionophore activity that inhibits the formation of the proton gradient vital for oxidative phosphorylation^19^. It has been reported that Cecropins inhibited the growth of harmful bacteria in the human digestive tract while maintaining a normal development of the intestinal flora^20^.

The use of peptides as antibiotics is challenging, as many of them show high sensitivity to proteases^21^. Indeed, most Cecropins present an abundant number of Lysine and Arginine amino acids, which are a constituent of many common recognition sequences for proteases, e.g. Proteinase-K, Inhibitor-A and Trypsin^22,23^. The design of bioengineered stable proteins has an important economic and technological value, as the limited stability of proteins severely restrains their therapeutic, veterinarian and industrial applications^14,24^.

In the following study, we present superior properties of an engineered AMP, OMN6, as well as applicability for future use as a life-saving treatment. OMN6, a 40-amino acid cyclic peptide based on Cecropin A, displays an increase in stability and a significant decrease in proteolytic degradation, together with a bactericidal effect on Gram-negative bacteria and a lack of cytotoxicity towards eukaryote cells. The target of OMN6 is assumed to be the bacterial membrane in contrast to small-molecule based agents which bind to a specific enzyme or bacterial site. OMN6 mechanism of action is effective on *Acinetobacter baumannii* laboratory strains and clinical isolates regardless of the bacteria genotype or resistance-phenotype, thus, is by orders-of-magnitude, less likely for mutation-driven development of resistance, recrudescence, or tolerance. It also presents full safety margin on erythrocytes and HEK293T cells. Taken together, these results strongly suggest that OMN6 is an efficient, stable, and non-toxic novel antimicrobial agent with the potential to become a therapy for humans.

## Methods

### Peptide synthesis

OMN6, a C-terminal amidated 40-amino acid long cyclic peptide with a molecular weight of 4339.36 Da, was synthetized as an acetate salt, using only l-amino acids, by Caslo ApS (Lyngby, Denmark; for stability, *E. coli* GFPuv, cytotoxicity, and hemolysis experiments) or Ambiopharm Inc. (North Augusta, SC, USA; for antimicrobial activity and time-kill studies). Here below are the details of a representative batch synthesis executed by Caslo ApS. Fmoc solid-phase peptide synthesis strategy on Rink Amide MBHA resin (loading capacity: 0.4 mmol/g) was used. Cyclization was achieved by oxidation in 50% DMSO/H_2_O under peptide concentration of 3 mg/mL. Cyclization success was assessed using ABI Voyager DE-PRO (ThermoFisher Scientific Corp., Waltham, MA, USA) Matrix Assisted Laser Desorption-Ionization Time of Flight Mass Spectrometry (MALDI-TOF-MS) with a matrix of 2,5-dihydroxybenzoic acid, and is presented in Figures S4 and S5. Peptide samples were purified by preparative reverse-phase high-performance liquid chromatography (RP-HPLC) using Agilent HP 1100 HPLC system (Agilent Technologies, Santa Clara, CA, USA) with an Ultimate C18 column (10 μm, 100 Å, 50 × 250 mm). A solvent system consisting of solvent A (0.1% TFA, 2% CH_3_CN in water) and solvent B (90% CH_3_CN/H_2_O) was used for elution with a gradient of 25–50% B in 60 min, and the absorbance was detected at 220 nm. Solvent was removed by lyophilization to afford a fluffy powder. The salt exchange from trifluoroacetate salt to acetate salt was also achieved by preparative RP-HPLC. The peptide was eluted by 0.02 M NH_4_Ac (approximate pH = 6) and solvent B. The elution solution containing target peptide was collected and then lyophilized. The purity (> 95%) of the purified material was assessed by analytical RP-HPLC with a Kromasil C18 column (4.6 × 250 mm), and the absorbance was detected at 220 nm.

### Modeling analysis

Exploratory studies of OMN6 structure were conducted by homology modeling from the helix-hinge-helix structure of papiliocin as was previously described and deposited in the Research Collaboratory for Structural Bioinformatics Protein Data Bank (RCSB PDB) under ID number 2LA2^25^. For modeling purposes, disulfide bond and structure refinement were estimated using the UCSF Chimera software^26^, as well as the PELE^27^, FG-MD^28^ and MRC^29^ web servers.

### Peptide stability

10 µg of synthetic cecropin A (Genscript, Piscataway, NJ, USA), OMN6, or bovine serum albumin (BSA; Sigma-Aldrich, St. Louis, MO, USA) were incubated with 5 or 20 ng Proteinase-K (Sigma-Aldrich; final treatment concentration of 0.16 or 0.66 µg/mL respectively) for 2 h at 37 °C, and separated on a 15% acrylamide gel (Bio-Rad, Hercules, CA, USA) by SDS-PAGE. The gel was then stained with Coomassie dye and excess color was removed over-night.

### Antimicrobial activity

This experiment was conducted by Omnix Medical, by JMI Laboratories (North Liberty, IA, USA) and by Aptuit (Verona) S.r.l. (Italy), an Evotec Company. All studies were performed according to Clinical and Laboratory Standards Institute guidelines^30^ for the application of the broth microdilution method. Reference strains from American Type Culture Collection (ATCC) and clinical isolates from the Evotec EvostrAIn and the JMI Laboratories SENTRY surveillance program culture collections (*Acinetobacter baumannii*: ACC 00445, ACC 00527, ACC 00535, ATCC 17978, ATCC 19606, ATCC BAA-1793, ACC 01076, ACC 01077, NCTC 13420, JMI #1088100 and JMI #1001007; *Klebsiella pneumoniae*: ATCC 43816 and ATCC BAA-1705; *Escherichia coli*: ACC 01001 and ATCC 25922; *Staphylococcus aureus*: ATCC 29213, ATCC BAA-44 and ATCC 33591; *Bacillus cereus:* ATCC 14579; *Enterococcus faecalis:* ATCC 29212; *Enterococcus faecium:* ATCC BAA-2319) were used. The antimicrobial activity of OMN6 was determined by 12 serial two-fold dilutions in water of the 100-fold highest compound solution; 1 µL of each well was transferred into 96 microtiter plates (final concentration: 256–0.12 µg/mL). In most strains, same procedure was used to determine the colistin antimicrobial activity (final concentration: 32–0.015 µg/mL). Bacterial strains were sub-cultured on the agar plates and incubated for 18–24 h at 35 ± 2 °C in ambient air. Following incubation, the inoculum was prepared from isolated colonies and adjusted to 0.5 McFarland turbidity standard (1 to 2 × 10^8^ colony-forming units/mL) in 0.9% saline. Bacterial suspension was then diluted 1:200 in Muller-Hinton broth (from BD; Becton, Dickinson and Company, Franklin Lakes, NJ, USA) and within 15 min 100 µL/well were dispensed. Growth-control wells and sterility-check wells (uninoculated well) were present in each plate. Plates were incubated for 18–24 h at 35 ± 2 °C in ambient air. The Minimum Inhibitory Concentration (MIC) value of each compound was determined as the lowest concentration that completely inhibits growth of the organism in microdilution well as detected by the unaided eye. The antimicrobial activity to other antibiotics on most bacterial strains was determined by Evotec using the antimicrobial sensitive test from the VITEK 2 automated microbiology system (bioMérieux SA, Marcy l'Etoile, France; data presented in Table S1), or by ATCC according to CLSI (Clinical and Laboratory Standards Institute) guidelines^30^ (data presented in Tables S2, S3).

### *E. coli* GFPuv experiments

*E. coli* GFPuv bacteria (contribution from Dr. Tsafi Danieli, head of the Protein Expression Facility at the Hebrew University of Jerusalem, Israel) were grown in LB broth (BD) and induced to express the Green Fluorescent Protein (GFP) in auto-induction medium, as previously described by F.W. Studier^31^. Briefly, bacteria were cultured for 3 h at 37 °C while shaking at 200 rpm, in induction medium ZYP-5052 containing 1% N-Z amine AS, 0.5% yeast extract, 50 mM Na_2_HPO_4_, 50 mM KH_2_HPO_4_, 25 mM (NH_4_)_2_SO_4_, 2 mM MgSO_4_, 0.5% glycerol, 0.05% glucose and 0.2% lactose. For the bacterium visualization experiment, 2 × 10^6^ colony-forming units (CFU) were treated by 16 µg/mL OMN6 or Double-Distilled Water (DDW) for 60 min. Bacteria were monitored with a confocal microscope at a wavelength of 488 nm. Then, bacteria were seeded on LB agar (BD) plates, incubated at 37 °C for 24 h, and CFU were counted. For the supernatant visualization and quantification experiment, 10^5^ CFU were co-incubated with 50 µM OMN6 or DDW for 30 min, and bacteria were collected and centrifuged at 5000 rpm for 10 min. Experiment tubes were visualized under UV light. Then, supernatants were collected, and the absence of a significant quantity of bacteria in the supernatants was checked in a microplate reader by absorbance at 600 nm (OD < 0.05). Fluorescence levels were then quantified with excitation at 395 nm and emission at 509 nm.

### Time-kill studies

Time-kill experiments were performed by JMI Laboratories (North Liberty, IA, USA) as described in chapter 5.14 of the Clinical Microbiology Procedures Handbook^32^ and the CLSI M26-A^33^ document. Two *Acinetobacter baumannii* clinical isolates (JMI colistin-sensitive strain #1088100 and colistin-resistant strain #1001007) were used for this study. MIC values that were determined in Difco Muller Hinton Broth (BD) for OMN6 and colistin as described above, were used as the baseline values during time-kill testing. In the time-kill experiments, OMN6 and colistin were tested in Difco Muller Hinton Broth. The time-kill cultures were sampled at 0, 2, 4, 8, and 24 h after compound addition. The number of CFU/mL in each culture was measured by plating 100 μL of serial dilutions onto blood agar plates. Colonies were counted after 48 h of growth at 35 °C. Time-kill curves were generated by plotting CFU/mL as log_10_ values over time. The limit of detection was 10 CFU/mL. Bactericidal activity was defined as a ≥ 99.9% reduction (i.e., a ≥ 3 log_10_ decrease) in the number of input bacterial cells at any arbitrary timepoint^33^.

### Cytotoxicity assay

HEK-293T17 cells (ATCC CRL-1573) were purchased from ATCC and cultured to 80% confluency according to the ATCC culture method, using high-glucose and l-Glutamine Dulbecco's Modified Eagle Medium (DMEM) medium (ATCC catalog number 30-2002) supplemented with 10% fetal bovine serum and 1% Penicillin–Streptomycin solution (all from Biological Industries). For the experiment purposes, 30,000 cells were transferred to a 96-well plate (experiment plate) in a final volume of 100 µL of medium. The experiment plate was incubated at 5% CO_2_ and 37 °C for approximately 18 h. After 18 h, medium was discarded, and cells were co-incubated with OMN6, Melittin (Sigma-Aldrich) or dimethyl sulfoxide (DMSO; MP Biomedical LLC, Solon, OH, USA) that were prepared in new medium at desired concentrations. The experiment plate was incubated at 5% CO_2_ and 37 °C for 24 h. Then, glutaraldehyde at a final concentration of 0.5% was added in all wells for 10 min for cell fixation. After washing the plate 3 times with DDW, the experiment plate was dried overnight. Then, cells were washed with 0.1 M Borate buffer (pH = 8.5) for 1 min. 100 µL Methylene Blue diluted in the same Borate buffer was added to all wells for 1 h. Wells were washed with DDW and plate was dried overnight. Color extraction was executed by adding 0.1 M HCl. After an incubation at 37 °C for 1 h, cell survival was determined via absorbance at 620 nm using a microplate reader.

### Hemolysis assay

Red Blood Cells (RBCs) suspension was prepared from whole blood extracted from the heart of Hsd:ICR (CD-1) mice (Envigo, Huntingdon, Cambridgeshire, UK). The study was carried out in accordance with relevant guidelines and regulations, and was approved by the Hebrew University Ethics Committee for the Care and Use of Laboratory Animals under approval number NS-15-14575-3. Briefly, blood was collected in a 24 U/mL heparin tube to prevent coagulation. Then cells were washed with Phosphate Buffered Saline (PBS; Biological Industries) and centrifuged at 200*g* for 10 min at 20 °C. This operation was repeated three times, then remaining RBCs were resuspended in saline or PBS to form a 10% RBC solution. Increasing concentrations of OMN6 or 1% Tween were added to RBCs. After an incubation of 1 h at 37 °C with shaking at 100 rpm, experiment tubes were centrifuged for 10 min at 200*g* at room temperature. 100 µL supernatant was extracted from each tube and tested according to the Hemoglobin Assay Kit (Sigma-Aldrich). Hemoglobin levels were determined via absorbance at 400 nm.

### Statistical analysis

All measurements were obtained at least three times and statistical analyses were performed using GraphPad Prism version 8.3 (San Diego, CA, USA). All data are presented as means ± SD from independent experiments. The statistical significance of differences between samples was analyzed by unpaired Student's t-tests. Differences with p < 0.05 were considered statistically significant.

## Results

### Peptide design and stability studies

**Figure 1:**
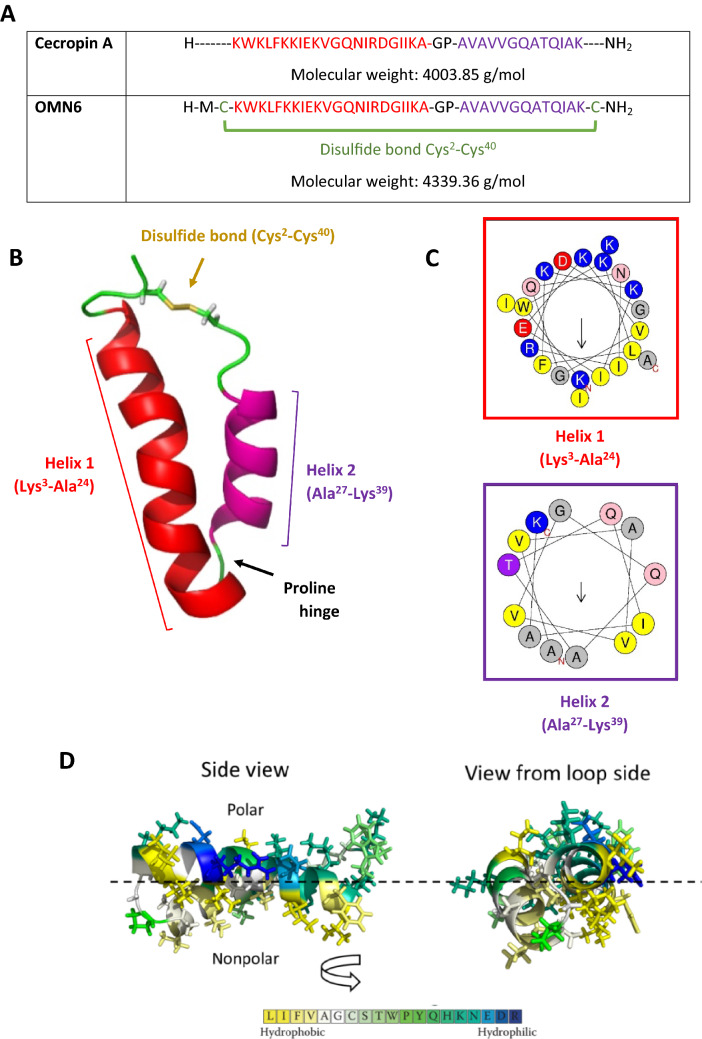

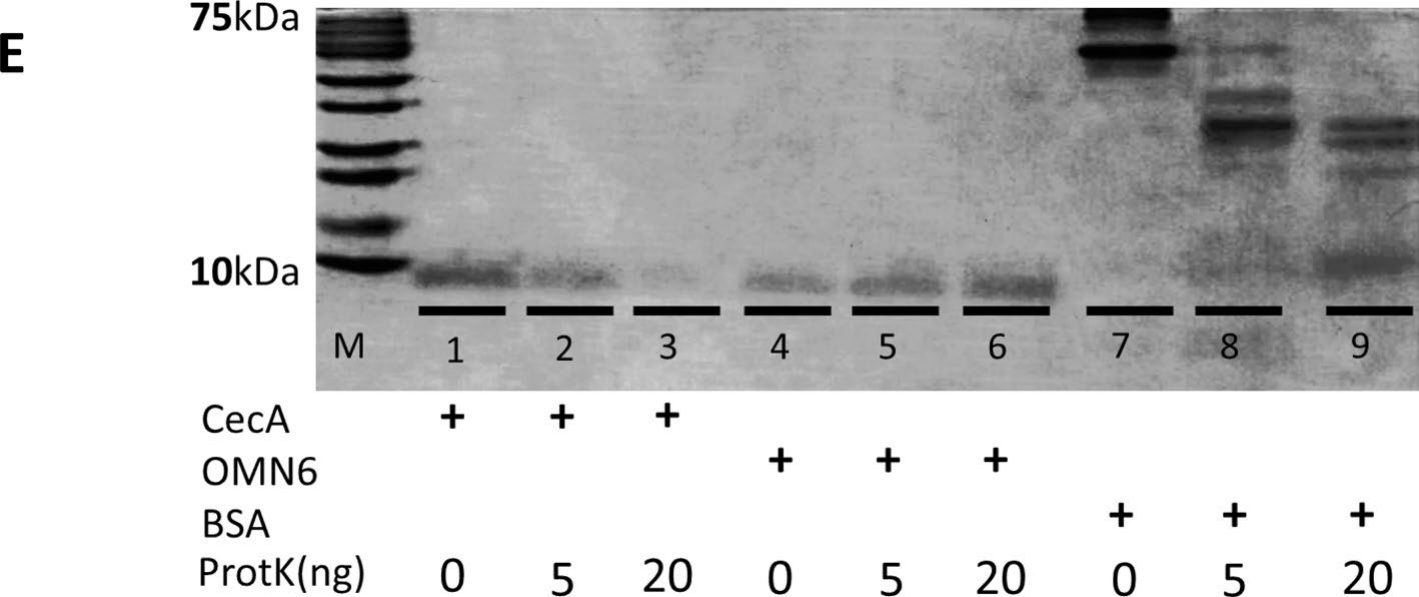
(**A**) OMN6 and Cecropin A amino-acid sequence comparison. Helical secondary structures are colored in red and purple. Disulfide bond between Cys^2^ and Cys^40^ is presented in green, the first helix in red, and the second one in purple. (**B**) Predicted structure of OMN6. The first helix is shown in red, the second one in purple, and the disulfide bond in yellow. (**C**) Helical wheel view of OMN6 generated by HeliQuest^68^. Blue: positively charged amino acids. Red: negatively charged amino acids. Pink: hydrophilic uncharged residues. Yellow: hydrophobic residues. Grey: l-Glycine and l-Alanine. Helical hydrophobic moment is displayed by the arrows. The N and C letters presented in red define the N-terminus and C-terminus ends of the α-helix structure. (**D**) Predicted three-dimension amphipathic nature of OMN6. Residues are colored by hydrophobicity (from yellow to blue). Dashed line, represents the boundary between nonpolar core of the membrane (bottom) and polar lipid headgroups region (top). (**E**) Western-blot of Cecropin A and OMN6 after exposure to Proteinase-K. Cecropin A is displayed in lanes 1–3, OMN6 in lanes 4–6, and Bovine Serum Albumin (positive control) in lanes 7–9.

The generation of new AMPs for therapeutic use was based on the native Cecropin family which serve as potent antimicrobial agents used by insects for millions of years. Bio-chemical engineering of our lead compound OMN6, a C-terminal amidated 40 amino-acid long cyclic peptide containing only l-amino acids, was based on the sequence of Cecropin A from the *Hyalophora cecropia* moth. l-Cysteines were incorporated at both the N- and C-terminal ends of the native linear peptide to instigate a disulfide bond formation. By this simple bridge formation, we yielded a cyclic peptide conformation that confers enhanced peptide stability in order to overcome the major hurdle in the development of AMPs as therapeutic agents^14,24^. Also, a l-Methionine amino acid was added at the N-terminal end (Fig. 1A). The goal of the l-Methionine addition was to enable potential peptide production by biomanufacturing using yeast or bacteria, as Methionine is the universal amino acid for initiation of protein synthesis in living organisms^34^. However, OMN6 has been produced so far only by chemical ways using solid-phase peptide synthesis, as the synthetic production process for OMN6 is robust and devoid of any endotoxins from bacterial source that may appear when using biosynthesis^35^. Predictions of OMN6 cyclic conformation were performed by modeling studies (Fig. 1B), according to previous structure studies on the Cecropin family^25^. In Fig. 1C, a helical wheel diagram of OMN6 shows the amphipathicity of the first α-helix (from Lys^3^ to Ala^24^) with hydrophobic residues in the upper region and hydrophilic residues in the lower region. The second α-helix (from Ala^27^ to Lys^39^) is hydrophobic and not amphipathic. As displayed in Fig. 1D, exploratory three-dimension modeling studies suggest that OMN6 has a polar side, in charge of the electrostatic attraction between the negatively charged head groups of membrane lipids and the positively charged amino acids of the peptide. Also, a non-polar side that interact with non-polar tails of membrane lipids are present in the OMN6 peptide.

In order to verify that the cyclization process did improve peptide stability, we compared the native peptide Cecropin A stability to that of OMN6 through Proteinase-K enzymatic degradation-based Western blot. Proteinase-K is a broad-spectrum serine protease that has high proteolytic activity. As shown in Fig. 1E, OMN6 remained intact after exposure to Proteinase-K, while Cecropin A was subjected to major degradation under the same conditions. This test confirms OMN6 increased biostability, when compared to the parent compound Cecropin A.

### Selective antimicrobial activity of OMN6

In order to assess OMN6 potential antimicrobial effect and range, we addressed several major global bacterial threats to Minimum Inhibitory Concentration (MIC) in vitro tests according to approved CLSI (Clinical and Laboratory Standards Institute) guidelines^30^. Gram (−) and Gram (+) ATCC strains as well as clinical isolates were selected. Gram (−) *Acinetobacter baumannii, Klebsiella pneumoniae, Escherichia coli* and Gram (+) *Staphylococcus aureus, Bacillus cereus, Enterococcus faecium* and *Enterococcus faecalis* were examined with many of the strains being multidrug resistant ones. As shown in Table 1, OMN6 presents a potent inhibitory effect on Gram-negative bacteria, with MIC of ≤ 32 µg/mL between the different tested bacterial strains, with no regard to their resistance profile. The lowest MIC observed was of the *A. baumannii* strains, with MICs ranging between 4–8 µg/mL, both in sensitive and multidrug resistant strains. OMN6 presented a MIC of ˃ 256 µg/mL on all tested Gram-positive strains—*S. aureus, E. faecium, E. faecalis* and *B. cereus*—showing a less than effective activity margin. All this gives the distinct indication that OMN6 retains an antimicrobial activity after the bioengineering steps required for stabilization have been performed. Thus, OMN6 presents powerful antimicrobial effect against Gram-negative bacteria and is active on *A. baumannii* regardless of any cross-resistance to other antibiotic drugs.

**Table 1:** OMN6 minimum inhibitory concentration results on 21 clinical isolates of Gram (−) and Gram (+) bacteria. Bacteria were subjected to Minimal Inhibitory Concentration (MIC) tests by broth microdilution method (CLSI guidelines). Resistance pattern of the different isolates is presented in the last column according to the following abbreviations: *MDR* multidrug resistant, *Col-S* colistin-sensitive, *Col-R* colistin-resistant, *Mer-S* meropenem sensitive, *Mer-R* meropenem resistant, *S* sensitive, *mcr-1* mobilized colistin resistance gene 1, *MSSA* methicillin-sensitive *Staphylococcus aureus*, *MRSA* methicillin-resistant *Staphylococcus aureus*, *Van-S* vancomycin sensitive, *Van-R* vancomycin resistant, *N/A* data not available. Supplementary information about the resistance pattern of most bacterial strains used in this study is displayed in Tables S1 and S2.

Bacteria	Strain	MIC (µg/mL)	Resistance pattern
*Acinetobacter baumannii* Gram (−)	ACC 00445	4	MDR/Col-S
	ACC 00527	4	MDR/Col-R
	ACC 00535	4	MDR/Col-S
	ATCC 17978	8	S
	ATCC 19606	8	S
	ACC 01076	8	MDR/Col-R
	ACC 01077	4	MDR/Col-R
	NCTC 13420	4	N/A
	ATCC BAA-1793	8	MDR
	JMI #1088100	8	Col-S/Mer-S
	JMI #1001007	4	Col-R/Mer-R
*Klebsiella pneumonia* Gram (−)	ATCC 43816	16	S
	ATCC BAA-1705	16	MDR
*Escherichia coli* Gram (−)	ATCC 25922	16	S
	ACC 01001	32	mcr-1
*Staphylococcus aureus* Gram (+)	ATCC 29213	> 256	MSSA
	ATCC 33591	> 256	MRSA
	ATCC BAA-44	> 256	MDR/MRSA
*Bacillus cereus* Gram (+)	ATCC 14579	> 256	N/A
*Enterococcus faecalis *Gram (+)	ATCC 29212	> 256	Van-S
*Enterococcus faecium* Gram (+)	ATCC BAA-2319	> 256	MDR/Van-R

### Mechanism of action

**Figure 2:**
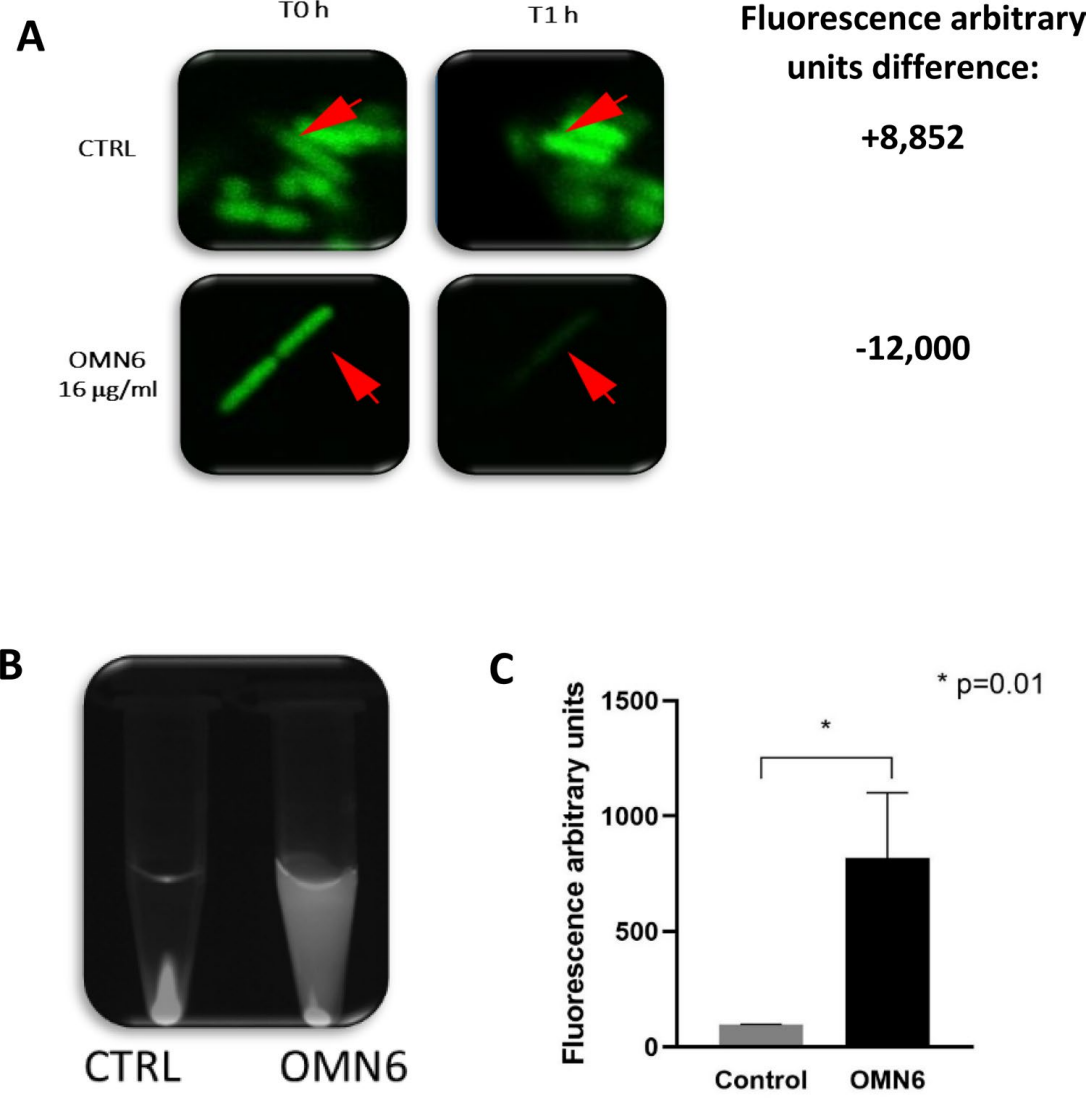
OMN6 mechanism of Action studies using the *E. coli* GFPuv model. (**A**) *E. coli* GFPuv bacteria were treated with double-distilled water (upper panels) or 16 µg/mL OMN6 (lower panels). Bacteria were monitored via confocal microscope at a 488 nm wavelength. During the experiment, levels of Florescence Units were continuously documented for a single bacterium (indicated by arrow). (**B**) *E. coli* GFPuv were treated with double-distilled water or 50 µM OMN6 and centrifuged at 5000 rpm after incubation. Experiment tubes were visualized under UV light. (**C**) Levels of fluorescence arbitrary units in the supernatants were quantified after a 96-well plate reader excitation at 395 nm and emission at 509 nm (*p = 0.01).

Membrane disruption is known as one of the characteristics of AMPs mechanism of action (MoA)^36^. This has led us to explore OMN6 induction of membrane disruption via fluorescence assays by utilizing the Gram (−) *E. coli* GFPuv construct bacteria as a model. As GFP is a large protein of 26.9 kDa, its exit or leakage out of the bacterial cell occurs only upon disruption of the bacterial membrane integrity. In this assay, *E. coli* GFPuv was incubated with 16 µg/mL OMN6 or double-distilled water (DDW) as the control group for one hour. As shown in Fig. 2A, an example of a bacterium in the control group was unaffected with no evidence of GFP leakage to the surrounding media, with an increase of 8,852 in fluorescence arbitrary units (FU) between T = 0 h to T = 1 h suggesting growth of the bacterial population. Bacteria treated with OMN6 displayed significant leakage of GFP out of the bacterial cell into the *milieu* showing a reduction of 12,000 in FU suggesting disruption of membrane integrity, as was observed with cecropins^37^ and other amphipathic peptides^38^. Bacterial death was confirmed at the end of each experiment by seeding all samples on Agar plates. No CFU were present in samples exposed to OMN6, where countless CFU were seen in the control samples (data not shown).

Next, we used *E. coli* GFPuv bacteria treated with DDW or OMN6 in order to visualize the bacteria under UV light. After the end of the treatment, we collected and centrifuged the bacterial samples in order to separate bacteria from media supernatant. In the control tube, fluorescence could be observed only in the pellet suggesting that GFP is restricted to the inside of the bacteria which are still intact. In the OMN6-treated tube, fluorescence was distributed in the supernatant and less bacterial cells were present in the pellet, suggesting GFP leakage has occurred (Fig. 2B). In Fig. 2C, we followed by separation of the supernatants from the pellets. After confirming by absorbance at 600 nm that no significant bacterial presence was present in the supernatants (OD < 0.05, data not shown), the level of fluorescence in the supernatants was quantified as FU. This showed a distinct increase of FU in the OMN6 treated group indicating again that OMN6 MoA causes the disruption of the bacterial membrane integrity.

### OMN6 presents a bactericidal effect on *Acinetobacter baumannii* colistin-sensitive and colistin-resistant strains

In order to determine the kinetics of bacterial killing induced by OMN6, time-kill assays were performed on two *A. baumannii* clinical isolates: the colistin-sensitive strain JMI #1088100, and the colistin-resistant strain JMI #1001007. This study was performed according to the CLSI approved guidelines^33^. Colistin was used as comparator in this study as it kills bacteria by membrane disruption^39^, and as it is used as a last-resort treatment in *A. baumannii* infections^40^. In order to establish the concentrations to be used in these time-kill assays, MIC of OMN6 and colistin were performed separately to the presented studies. As shown in Table 1, MIC OMN6 value was of 8 µg/mL for the JMI #1088100 colistin-sensitive strain and of 4 µg/mL for the JMI #1001007 colistin-resistant strain. By comparison, MIC colistin value was of 0.5 µg/mL for the JMI #1088100 colistin-sensitive strain and of 8 µg/mL for the JMI #1001007 colistin-resistant strain (data not shown).

**Figure 3:**
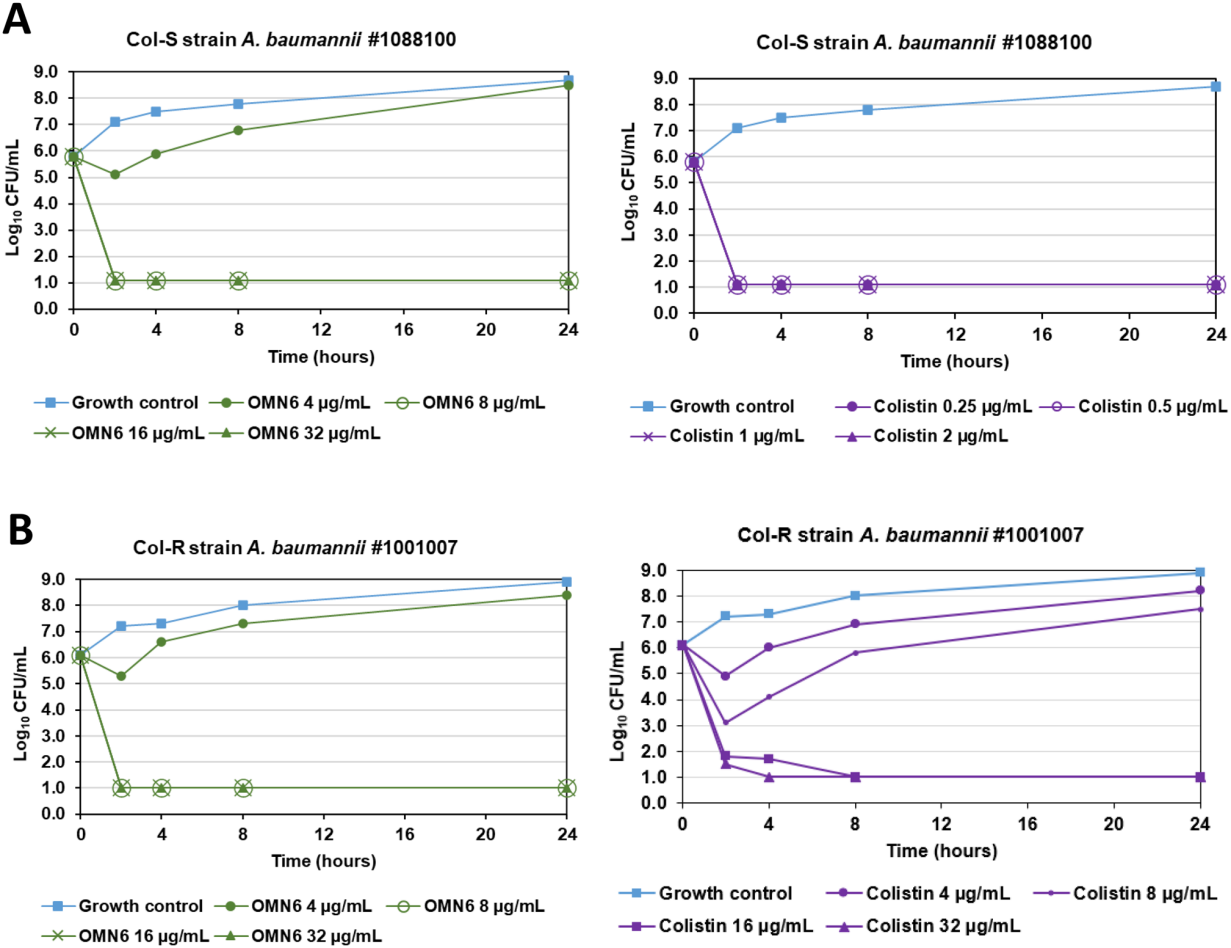
Fast bactericidal effect of OMN6 on colistin-sensitive and colistin-resistant *A. baumannii* strains. (**A**) Comparison of OMN6 (left) and colistin (right) killing kinetics on colistin-sensitive *A. baumannii* JMI #1088100 strain. (**B**) Comparison of OMN6 (left) and colistin (right) killing kinetics on colistin-resistant *A. baumannii* JMI #1001007 strain. Quantity of bacteria is displayed in log_10_ CFU/mL.

Figure 3A presents the time course of killing of the colistin-sensitive *A. baumannii* JMI #1088100 strain by OMN6 and colistin. Both agents showed a bactericidal effect after 2 h of incubation at concentrations superior to 8 µg/mL for OMN6 and 0.25 µg/mL for colistin. No re-growth was observed during the 24 h timeframe. However, on the colistin-resistant strain presented in Fig. 3B, while colistin showed a bactericidal effect only at high concentrations of 16–32 µg/mL, OMN6 retained its bactericidal pattern with similar timeframe and concentrations. To conclude, this experiment shows that OMN6 and colistin act on the same timeframe, but that OMN6 is superior to colistin as it stays active even on colistin-resistant strains.

### Cytotoxicity and hemolytic effect

**Figure 4:**
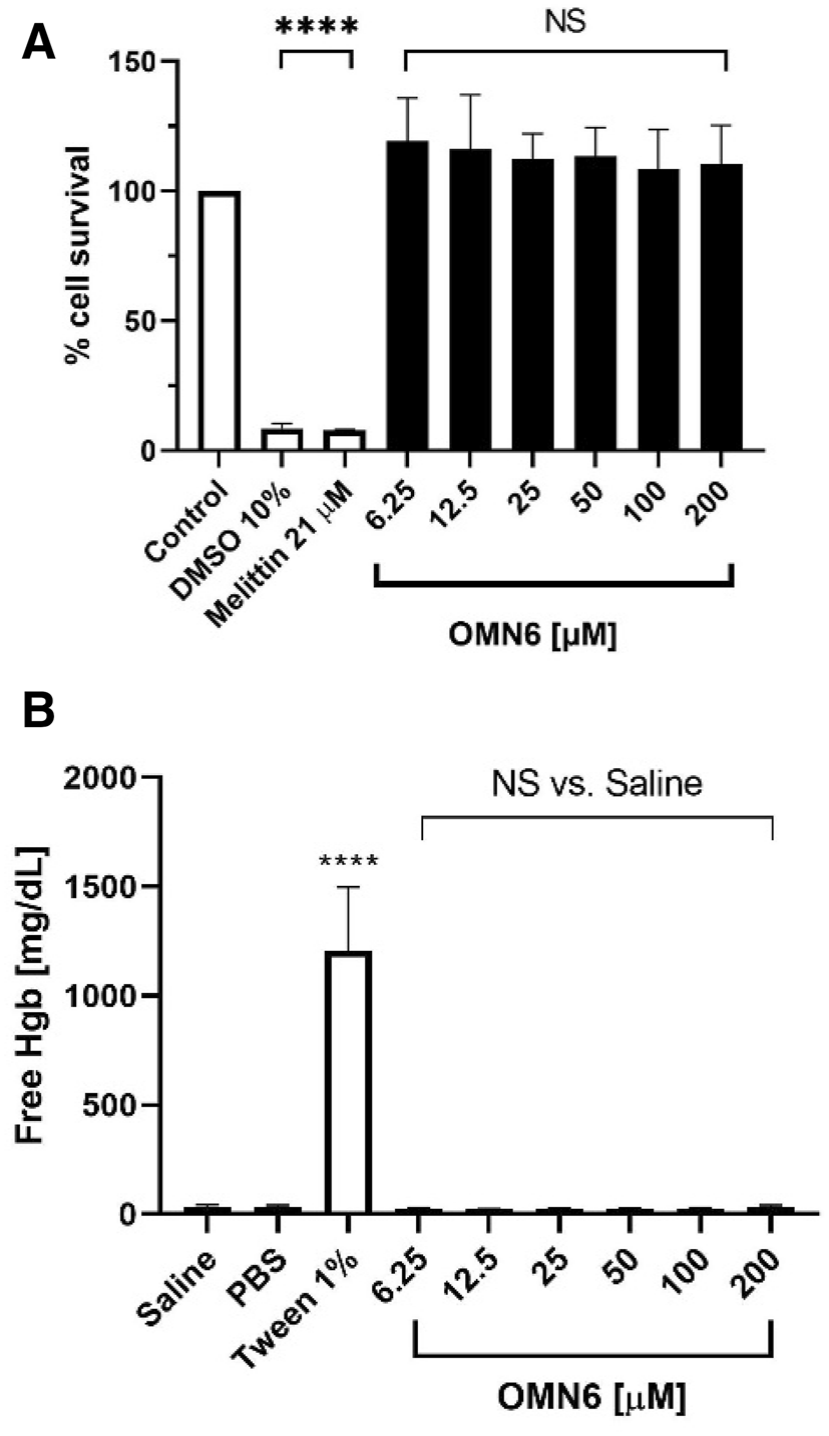
OMN6 demonstrates no cytotoxicity or hemolytic effect on mammalian cells. (**A**) Cytotoxicity assay via HEK293T cells in vitro. HEK293T cells were exposed to increasing concentrations of OMN6, while the control group was treated with double distilled water (DDW). Positive control groups were exposed to 10% DMSO or 21 µM melittin, that caused > 90% cytotoxicity. After 24 h, all experiment groups were subjected to Methylene-Blue assay in order to evaluate cell survival in comparison to the control group at 100%. (*NS* not significant; ****p < 0.0001). (**B**) Hemolysis assay via mouse red blood cells. Suspensions of 10% mouse erythrocytes were exposed to increasing concentrations of OMN6. The positive control group was treated with 1% Tween. The negative control group for the peptide treatment and for the Tween treatment were respectively treated with 0.9% saline solution and with PBS × 1. After a one-hour incubation, the relative amount of free hemoglobin (Hgb) was assessed as an indication of erythrocyte hemolysis (*NS* not significant; ****p < 0.0001).

As OMN6 targets membranes, it is vital to determine, as soon as possible, whether the activity of membrane permeability is selective to bacterial cells and will not affect mammalian cells. To this end, investigative in vitro safety studies were conducted. Firstly, we tested OMN6 for the presence of a cytotoxic effect on HEK293T embryonic cell-line. In this study, HEK293T cells were exposed to increasing concentrations of OMN6 (6.25–200 µM, equivalent to 27–868 µg/mL). Melittin, a 26-amino acid basic peptide that is the major pain producing substance of the honeybee (*Apis mellifera*) venom^41^, was used as a positive control in this experiment, as it is a non-selective antimicrobial peptide that targets mammalian membranes and bacterial membranes alike^18,42^. As illustrated in Fig. 4A, the survival rate of HEK293T cells was not affected by the presence of the peptide at all concentrations. Moreover, no significant changes in cell death, cell morphology and alteration in survival fractions could be observed in any of the OMN6 groups. On the other hand, more than 90% cell death was observed on cells exposed to 21 μM melittin (equivalent to 60 μg/mL, within the effective range of activity of melittin).

Then, the hemolytic effect of OMN6 was assessed by exposure of mouse erythrocytes to similar concentrations of peptide (ranging between 6.25–200 µM, equivalent to 27–868 µg/mL). Tween at 1% caused massive hemolysis and as shown in Fig. 4B, no hemolysis expressed as free Hemoglobin was detected in all OMN6 exposed groups. Together, the observations made here show that the novel AMP OMN6 is a selective new antimicrobial agent presenting high stability and bacteriolytic activity, with no toxicity to mammalian cells even at high doses, thus rendering it acceptable for further development as a therapeutic solution against resistant bacteria.

## Discussion

Antimicrobial peptides (AMPs) are a family of potent innate immunity effectors. In insects, Cecropins form a large family of cationic α-helical AMPs with wide antimicrobial and anti-inflammatory effects^10,14,15,17,25,43–45^. Cecropins cause membrane disruption by a unique mode of action that has been described by Gazit et al.^37^, it involves initial adsorption of the peptide onto the membrane surface, driven by the electrostatic attraction between the negatively charged head-groups of bacterial membrane lipids and the positively charged amino acids of the peptide. This external interaction is followed by rearrangement of the peptide orientation, so its nonpolar amino acids face the core of the membrane, where they can favorably interact with the nonpolar tails of membrane lipids. When a sufficient number of peptides bind to the membrane, they apply a strain on the membrane that results in its disintegration. This mode of action requires the peptide to be sufficiently amphipathic to be attracted by the bacterial membrane, and hydrophobic enough to partially penetrate the membrane surface and interact with the core.

In the current study, we present OMN6, a new cyclic antimicrobial peptide based on the unique family of Cecropins isolated from the innate immune systems of the *Hyalophora cecropia* moth. By using initial modeling studies, we observed that the peptide was amphipathic presenting a polar side which includes positively charged amino acids, and a nonpolar hydrophobic side. These preliminary modeling studies results agree with the literature regarding the structure of the parental peptide Cecropin A^43,46^. These results will need to be confirmed by other techniques—such as Nuclear Magnetic Resonance (NMR) spectroscopy or X-ray crystallography—to fully assess the three-dimensional structure of OMN6.

The cyclized form of OMN6 confers high stability to the compound and prevents its proteolytic degradation by commonly present proteases, while upholding its full bioactivity. Our results corroborate with other teams' observations that showed disulfide bridges confer high stability to natural cyclic peptides from the plant defensins or the cyclotide families^47,48^. Likewise, an increased stability was obtained on linear AMPs after their cyclization, while keeping the peptide antimicrobial properties^49–51^.

Our findings display OMN6 efficient antimicrobial activity against Gram-negative bacteria, including against the concerning ESKAPE pathogens that cause life-threatening nosocomial infections^52^.

At the top of the list of most urgent infectious threat as defined by the CDC^3^ and the WHO^53^ stands carbapenem-resistant *Acinetobacter baumannii*. We presented OMN6 ability to eliminate all clinical isolates of *A. baumannii* whether these strains were sensitive or multidrug resistant to commonly used antimicrobial drugs. In vitro OMN6 superiority on colistin was also assessed by time-kill curve studies in which OMN6 presented a bactericidal effect, with a similar pattern observed on a colistin-sensitive and on a colistin-resistant *A. baumannii* strain. The promising in vitro profile presented by OMN6 in our studies will need to be confirmed in vivo, as colistin, that is used as a last resort treatment in *A. baumannii*-based infections^40^, presents high nephrotoxicity and needs to be urgently replaced by safer therapies^54^.

When investigating the in vitro activity of OMN6 against bacterial species, we observed membrane disruption features, with leakage of internal bacterial components to the external media. These results are consistent with the known effects of cecropins and similar membrane-disrupting AMPs, as has been previously described in scientific literature^16,55–58^. This distinctive mode of action by membrane disruption bestows key advantages to the AMPs family. Unlike most antibiotics that are dependent on a biochemical specific site of action or interaction, AMPs exert a physical damage on the actual structure of the whole bacterial membrane^14,15^. This mechanism has low propensity to develop antimicrobial resistance both during and after a treatment^59^. Taken together, our results with the previous literature suggest that OMN6 causes membrane disruption and bacterial lysis and death. Though, the precise sequence of events leading to bacterial death after exposition to OMN6 remains to be fully elucidated.

Another important characteristic of the AMPs MoA is their specificity to well organized negatively charged membranes which differentiate bacteria from eukaryotic cells^60^. Our investigations showed that OMN6 presented no toxicity to mammalian cells, even at high concentrations. This therapeutic window, that is larger than tenfold of the MIC value against Gram-negative bacterial species, allows OMN6 to become a therapy for humans.

The selectivity of OMN6 to bacterial membranes is assumed to derive from the peptide net positive charge and from it adopting a biologically active conformation contingent only upon binding to negatively charged bacterial membranes. By contrast, we presume that eukaryote membranes were not targeted by OMN6, since they are nearly electrostatically neutral^61^ and present high amounts of cholesterol causing resistance to membrane perturbation^57^. This parameter is not the single one predicting OMN6 effects on bacteria, as Gram-positive bacteria are also negatively charged^62,63^ and OMN6 showed no activity against all Gram-positive bacterial strains (*Staphylococcus aureus*, *Bacillus cereus*, *Enterococcus faecalis* and *Enterococcus faecium*) tested in this study. We assume, as reviewed by Malanovic et al.^64^, that additional membrane features and components may have effects on AMP activity, which could explain the different effects observed with OMN6 on Gram-positive and Gram-negative bacteria. For example, Gram-positive bacteria have a thick peptidoglycan-based cell wall containing lipoteichoic acid that may play as AMP entrapper, resulting in a decrease in local peptide concentration on the cytoplasmic membrane, and finally in protection from membrane disruption caused by AMPs^64,65^. These results would explain why OMN6, like Cecropins A and B^25,44,66,67^, have a better antimicrobial effect on Gram-negative bacteria than on Gram-positive bacteria.

## Conclusion

Collectively, our data shows that the cecropin-based novel peptide, OMN6, presents high activity against drug resistant Gram-negative bacteria, employing a bactericidal mechanism of action. OMN6 enhanced resistance to proteolysis and lack of toxicity toward eukaryote mammalian cells, make this novel peptide druggable as well as efficient. All these properties make OMN6 a perfect candidate to be developed as the next generation of anti-infective therapies.

## Supplementary Information

Supplementary Information.
