# Supplementary material for: OMN6 a novel bioengineered peptide for the treatment of multidrug resistant Gram negative bacteria
Source: Sci Rep. 2021 Mar 23;11:6603. doi: 10.1038/s41598-021-86155-9 (PMC7988117; doi:10.1038/s41598-021-86155-9)
Supplement: Supplementary file 1 — Supplementary Information. [file 41598_2021_86155_MOESM1_ESM.docx]

**TITLE**

OMN6 a Novel Bioengineered Peptide for the Treatment of Multidrug Resistant Gram Negative Bacteria

**AUTHORS**

Shira Mandel 1, Janna Michaeli 1, Noa Nur 1, Isabelle Erbetti 2, Jonathan Zazoun 1, Livia Ferrari 2, Antonio Felici 2, Moshe Cohen-Kutner 1, Niv Bachnoff* 1

**AFFILIATIONS**

1 Omnix Medical Ltd., High-Tech village, Givat-Ram Campus, 9270401 Jerusalem, Israel

2 Evotec Anti-infective, Department of Microbiology Discovery, Aptuit (Verona) S.r.l., *an Evotec Company*, via A. Fleming 4, 37135 Verona, Italy

*Corresponding author. E-mail: [niv@omnixmedical.com](mailto:niv@omnixmedical.com)

**Table S1. Supplementary information about the resistance pattern of 14 bacterial strains used in the Minimum Inhibitory Concentration study presented in Table 1.** The resistance pattern of the different bacterial isolates was characterized by broth microdilution studies (for colistin) or with the VITEK 2 system (for the other antibiotics). The resistance range of each antibiotic has been determined according to CLSI guidelines, and is presented in the next table according to the following abbreviations: S, sensitive; I, intermediate; R, resistant; GEN, gentamycin; AUG, amoxicillin/clavulanic acid; IMP, imipenem; CTX, cefotaxime; CIP, ciprofloxacin; PTZ, piperacillin/tazobactam; CAZ, ceftazidime; CLI, clindamycin; ERY, erythromycin; TET, tetracycline; VAN, vancomycin; OXA, oxacillin; MDR, multidrug resistant; Col-S, colistin-sensitive; Col-R, colistin-resistant; S, sensitive; mcr-1, mobilized colistin resistance gene 1; MSSA, methicillin-sensitive *Staphylococcus aureus*; MRSA, methicillin-resistant *Staphylococcus aureus;* N/A, data not available.

|  |  |  |  | **VITEK characterization** | | | | | | |
| --- | --- | --- | --- | --- | --- | --- | --- | --- | --- | --- |
| **Bacteria** | **Strain** | **Resistance pattern** | **Colistin MIC (µg/mL)** | **GEN** | **AUG** | **IMP** | **CTX** | **CIP** | **PTZ** | **CAZ** |
| *Acinetobacter baumannii Gram (-)* | ACC 00445 | MDR/Col-S | 2 | R | R | R | R | R | R | R |
|  | ACC 00527 | MDR/Col-R | 32 | R | R | R | R | R | R | R |
|  | ACC 00535 | MDR/Col-S | 0.25 | R | R | I | R | R | R | R |
|  | ATCC 17978 | S | 0.25 | S | R | S | S | S | S | S |
|  | ATCC 19606 | S | 0.5 | I | R | S | I | S | S | I |
|  | ACC 01076 | MDR/Col-R | >32 | R |  | R |  | R |  |  |
|  | ACC 01077 | MDR/Col-R | 8 | R |  | R |  | R |  |  |
|  | NCTC 13420 | N/A | 0.25 | R |  | I |  | R |  |  |
| *Klebsiella pneumoniae Gram (-)* | ATCC 43816 | S | 0.25 | S | S | S | S | S | S | S |
|  | ATCC BAA-1705 | MDR | 0.5 | S | R | R | R | R | R | R |
| *Escherichia coli Gram (-)* | ACC 01001 | mcr-1 | 2 | S | S | S | S | S | S | S |
|  | ATCC 25922 | S | 0.25 | S | S | S | S | S | S | S |
|  |  |  |  |  | | | | | | |
|  |  |  |  | **VITEK characterization** | | | | | | |
| **Bacteria** | **Strain** | **Resistance pattern** | **Colistin MIC (µg/mL)** | **CLI** | **IMP** | **CIP** | **ERY** | **TET** | **VAN** | **OXA** |
| *Staphylococcus aureus Gram (+)* | ATCC 29213 | MSSA | >32 | S | S | S | S | S | S | S |
|  | ATCC 33591 | MRSA | >32 | R | R | S | R | R | S | R |

**Table S2. Supplementary information about the resistance pattern of the bacterial strain *E. faecium* BAA-2319 used in the Minimum Inhibitory Concentration study presented in Table 1.** The resistance range of each antibiotic has been determined by ATCC according to CLSI guidelines, and is presented in the next table according to the following abbreviations: S, sensitive; R, resistant.

| Antibiotic name | Resistance pattern |
| --- | --- |
| Ampicillin | R |
| Benzylpenicillin | R |
| Ciprofloxacin | R |
| Erythromycin | R |
| Levofloxacin | R |
| Nitrofurantoin | R |
| Quinupristin/Dalfopristin | R |
| Tetracycline | R |
| Tigecycline | S |
| Vancomycin | R |

**Table S3. Supplementary information about the resistance pattern of the bacterial strain *A. baumannii* BAA-1793 used in the Minimum Inhibitory Concentration study presented in Table 1.** The resistance range of each antibiotic has been determined by ATCC according to CLSI guidelines, and is presented in the next table according to the following abbreviations: S, sensitive; R, resistant.

| Antibiotic name | Resistance pattern |
| --- | --- |
| Amikacin | S |
| Amoxicillin / Clavulanic acid | R |
| Ampicillin | R |
| Ampicillin/Sulbactam | S |
| Aztreonam | R |
| Cefalotin | R |
| Cefazolin | R |
| Cefepime | R |
| Cefotaxime | R |
| Cefotetan | R |
| Cefoxitin | R |
| Cefpodoxime | R |
| Ceftazidime | R |
| Ceftizoxime | R |
| Ceftriaxone | R |
| Cefuroxime | R |
| Cefuroxime Axetil | R |
| Ciprofloxacin | R |
| Gentamicin | S |
| Imipenem | R |
| Levofloxacin | I |
| Meropenem | R |
| Moxifloxacin | R |
| Nalidixic Acid | R |
| Nitrofurantoin | R |
| Norfloxacin | R |
| Piperacillin | R |
| Piperacillin / Tazobactam | R |
| Tetracycline | R |
| Ticarcillin | R |
| Ticarcillin / Clavulanic acid | R |
| Tigecycline | S |
| Tobramycin | R |
| Trimethoprim / Sulfamethoxazole | S |

**Figure S4. MALDI-TOF-MS spectrum of the linear OMN6.** The measured mass of the [M+H]^+^ molecular ion at m/z 4343.13 is consistent with the molecular weight of linear OMN6 (4341.36 Da).

**Figure S5. MALDI-TOF-MS spectrum of the cyclic OMN6.** The measured mass of the [M+H]^+^ molecular ion at m/z 4340.73 is consistent with the molecular weight of cyclic OMN6 (4339.36 Da).
